# Functional Relationship between Leptin and Nitric Oxide in Metabolism

**DOI:** 10.3390/nu11092129

**Published:** 2019-09-06

**Authors:** Sara Becerril, Amaia Rodríguez, Victoria Catalán, Beatriz Ramírez, Xabier Unamuno, Piero Portincasa, Javier Gómez-Ambrosi, Gema Frühbeck

**Affiliations:** 1 Metabolic Research Laboratory, Clínica Universidad de Navarra, 31008 Pamplona, Spain (A.R.) (V.C.) (B.R.) (X.U.) (J.G.-A.) (G.F.); 2 CIBER Fisiopatología de la Obesidad y Nutrición (CIBEROBN), Instituto de Salud Carlos III, 31008 Pamplona, Spain; 3 Obesity and Adipobiology Group, Instituto de Investigación Sanitaria de Navarra (IdiSNA), 31008 Pamplona, Spain; 4 Medical Engineering Laboratory, University of Navarra, 31008 Pamplona, Spain; 5 Clinica Medica “A. Murri”, Department of Biomedical Sciences and Human Oncology, University of Bari Medical School, Policlinico Hospital, 70124 Bari, Italy; 6 Department of Endocrinology & Nutrition, Clínica Universidad de Navarra, 31008 Pamplona, Spain

**Keywords:** leptin, nitric oxide, nitric oxide synthase

## Abstract

Leptin, the product of the *ob* gene, was originally described as a satiety factor, playing a crucial role in the control of body weight. Nevertheless, the wide distribution of leptin receptors in peripheral tissues supports that leptin exerts pleiotropic biological effects, consisting of the modulation of numerous processes including thermogenesis, reproduction, angiogenesis, hematopoiesis, osteogenesis, neuroendocrine, and immune functions as well as arterial pressure control. Nitric oxide (NO) is a free radical synthesized from L-arginine by the action of the NO synthase (NOS) enzyme. Three NOS isoforms have been identified: the neuronal NOS (nNOS) and endothelial NOS (eNOS) constitutive isoforms, and the inducible NOS (iNOS). NO mediates multiple biological effects in a variety of physiological systems such as energy balance, blood pressure, reproduction, immune response, or reproduction. Leptin and NO on their own participate in multiple common physiological processes, with a functional relationship between both factors having been identified. The present review describes the functional relationship between leptin and NO in different physiological processes.

## 1. Introduction

### 1.1. Leptin

The discovery in 1994 of leptin, the product of the *ob* gene [1], was a turning point in modern physiology. Traditionally, adipose tissue (AT) was considered as a passive organ for energy storage, but this view was overturned by the identification of leptin as an adipocyte-derived hormone crucial for the control of feeding, metabolism, and body weight, being nowadays considered as an endocrine organ that secretes a wide variety of hormones and metabolically active substances [2]. Leptin is a 16-kDa protein that exhibits striking structural similarities to members of the long-chain helical cytokine family such as interleukin (IL)-6, IL-2, IL-12, or leukocyte inhibitory factor (LIF) [3]. The leptin receptor (OB-R or LEPR) belongs to the class I cytokine receptor family and six isoforms (OB-Ra-OB-Rf) are produced by alternative splicing with different intracellular lengths or ectopic shedding [one long form (OB-Rb), four short forms (OB-Ra, c, d, and f), and the soluble receptor, OB-Re] [4]. The full-length isoform OB-Rb contains the intracellular motifs required for the activation of the Janus kinase/signal transducer and activator of the transcription (JAK/STAT) pathway, one of the main transduction signals whereby leptin exerts its physiological actions. Leptin is mainly produced and secreted by adipocytes in proportion to fat stores, but the skeletal muscle, intestine, brain, placenta and bone, among others, are also able to release this hormone [5,6]. Leptin is mainly involved in metabolic homeostasis, which can be attained by the delivery of information about the amount of energy stores to the hypothalamus that in turn alters the central nervous system (CNS) function and regulates the synthesis of glucocorticoids and insulin as well as food intake and energy expenditure. Nevertheless, leptin is not only an AT-derived messenger of the amount of energy stores to the brain, but is also a crucial cytokine for a number of diverse physiological processes including inflammation, angiogenesis, hematopoiesis, reproduction, and immune function through its effects on peripheral tissues [7].

### 1.2. Nitric Oxide

Nitric oxide (NO) is a liposoluble free radical with a short half-life that functions as a soluble messenger in numerous tissues [8]. NO was first identified as the key endothelium-derived molecule promoting vasodilation and it was originally termed as endothelial-derived relaxing factor (EDRF) [9]. NO has multiple biological actions, regulating physiology acutely or leading to long-term changes in cell function. The pleiotropic roles of NO include the regulation of long-term synaptic transmission, reproduction, memory, platelet aggregation, leukocyte-endothelial interactions, immune function, and angiogenesis, among others. NO is produced from L-arginine and oxygen in a reaction catalyzed by three isoforms of the enzyme nitric oxide synthase (NOS): neuronal NOS (nNOS or NOS-1), endothelial NOS (eNOS or NOS-2), and inducible NOS (iNOS or NOS-3). Both nNOS and eNOS are constitutively synthesized under physiological conditions, and are mainly expressed in neurons and endothelial cells, respectively. They are calcium-dependent and produce low levels of NO. In a marked contrast, the expression of the inducible and calcium-independent iNOS in healthy conditions is absent, being strongly upregulated under pathophysiological conditions and producing high levels of NO [10]. The different isoforms of NOS have diverse physiological functions. NO produced in vascular endothelial cells acts in smooth muscle cells activating the soluble guanylyl-cyclase, thus, leading to vasodilation. NO produced by nNOS acts as an atypical neurotransmitter whereas the high amounts of NO generated by iNOS mediate an inflammatory response. The important role of NOS in whole-body homeostasis has been revealed in triple nNOS/eNOS/iNOS-null mice that exhibit renal (nephrogenic diabetes insipidus and pathological renal remodeling), lung (accelerated pulmonary fibrosis), and bone (increased bone mineral density and bone turnover) abnormalities [11]. 

The oxygen independent pathway nitrate–nitrite–nitric oxide for NO generation represents an important alternative source of NO to the classical L–arginine–NO-synthase pathway, particularly in hypoxic states [12]. Dietary nitrate is reduced, via facultative anaerobic bacteria inhabiting the gastrointestinal tract, to NO during reduced oxygen availability conditions such as altitude or pathologies (coronary artery disease or heart failure, among others), when NOS expression is limited. A robust NO-like bioactivity after the ingestion of nitrate has been demonstrated, leading to a reduction in blood pressure [13], increased mitochondrial efficiency [14], inhibition of platelet aggregation [15], improved vascular function [16], enhanced FA oxidation in skeletal muscle [17] as well as beneficial effects on diabetes [18].

The first study that revealed that leptin could induce NO production was the work of Yu et al.; providing evidence that leptin acts at both the hypothalamic and pituitary level to stimulate NO release [19]. Wang et al. thereafter confirmed this relationship in pancreatic islets [20]. Leptin administration increases NO production in a dose-dependent manner by activating NOS [21]. Furthermore, it has been reported that the circadian rhythm of both molecules fluctuate in parallel [22]. No differences in plasma leptin levels are observed after long-term dietary nitrate supplementation, suggesting that the oxygen-independent pathway is not impacted by this adipokine. The present review describes the functional relationship between leptin and NO in different physiological processes

## 2. Energy Balance

### 2.1. Leptin and Energy Balance

#### 2.1.1. Food Intake and Body Weight

Leptin is released into the blood and circulates to the brain where it crosses the blood–brain barrier via a saturable process, acting at leptin receptors within the CNS, and affecting appetite and thermogenesis as well as body weight, among others. The LepR is expressed in the arcuate nucleus (ARC) and in other nuclei of the mediobasal hypothalamus, where it modulates the homeostatic control of feeding and energy expenditure. In addition, the LepR is also expressed in other brain areas including the lateral hypothalamus and the ventral tegmental area (VTA), where leptin controls food reward [23].

Hypothalamic neuropeptides involved in leptin action have been classified into two major groups. The orexigenic peptides including neuropeptide Y (NPY), agouti gene-related protein (AgRP), melanin-concentrating hormone (MCH), and orexins stimulate appetite, are inhibited by leptin, and increase in response to leptin deficiency. The anorexigenic peptides, which inhibit feeding, are stimulated by leptin and decrease in response to leptin deficiency, and include α-MSH (derived from proopiomelanocortin POMC), cocaine-amphetamine-regulated-transcript (CART), and corticotropin-releasing hormone (CRH). Furthermore, the paraventricular nucleus (PVN) transduces leptin signaling during periods of changing energy availability, being involved in the maintenance of energy homeostasis and feeding behavior, and contributing significantly to leptin’s effects. Lesions of the PVN have been shown to induce hyperphagia and obesity [24]. The intracerebroventricular (ICV) administration of leptin decreased cumulative food intake and body weight in leptin deficient *ob*/*ob* mice [25], confirming that the CNS is a main target for leptin actions. 

The leptin–melanocortin pathway exhibits a strong influence on food intake and energy expenditure [26]. Leptin inhibits the neuropeptide Y/agouti-related protein (NPY/AgRP) production and stimulates pro-opiomelanocortin (POMC) production. POMC-containing neurons (located in the ARC) produce α-and β-melanocyte-stimulating hormone (α-and β-MSH) via the processing of prohormone convertase 1 (PC1/3) and carboxypeptidase E (CPE) enzymes. α-and β-MSH bind to melanocortin 3 and melanocortin 4 receptors (MC3R and MC4R) and induce their activity. Mutations in the leptin/melanocortin pathway (*OB, LEPR, POMC, PCK1, MC4R*) are associated with obesity in rodents and humans [27,28].

#### 2.1.2. Thermogenesis

Leptin plays an important physiological role in the regulation of thermogenesis, increasing energy expenditure via the activation of the sympathetic nerve activity and the turnover of norepinephrine in brown AT (BAT), the fat depot specializing in heat production. Leptin stimulates β_3_-adrenergic receptors (β_3_-AR), thereby leading to increased thermogenesis through the upregulation of the expression levels of the peroxisome proliferator-activated receptor γ coactivator-1 α (PGC-1α) and the uncoupling proteins 1 and 3 (UCP-1 and UCP-3) [29,30,31]. In line with this observation, leptin-deficient *ob*/*ob* mice exhibit a decreased energy expenditure, an impaired thermogenic BAT function and morphology together with lower transcript levels of *Ucp1* and *Ucp3* in BAT [32]. Accordingly, ICV administration of leptin increases metabolic rates in *ob*/*ob* mice [33].

The presence of brown-like cells within WAT termed beige or brite (brown-in-white) adipocytes has been described [34]. Beige adipocytes exhibit a unique gene signature and upon prolonged β-adrenergic stimulation such as cold exposure or exercise, beige fat cells express high amounts of UCP-1 and acquire thermogenic properties. Central leptin administration activates WAT differentiation toward a BAT-like phenotype through the activation of the CNS [35]. Moreover, leptin directly acts on murine subcutaneous adipocytes upregulating transcript levels of *Prdm16*, a zinc finger transcription factor controlling the bidirectional cell fate switch between myoblasts and brown adipocytes required for the browning of white fat as well as UCP-1 [36,37]. Moreover, leptin activates the JAK2/STAT3 pathway in adipocytes, with STAT3 interacting with PRDM16 to promote fat browning [38]. 

Exercise induces profound adaptations on brown and beige adipose tissue such as an increase in mitochondrial activity, decrease in adipocyte cell size, and lipid content or regulation of adipokines, which contribute to the improvement of metabolic health [39,40]. The discovery of factors secreted by the contracting muscle has revealed that these molecules, termed myokines, can act on adipocytes as positive (IL-6, irisin, BAIBA and meteorin-like) and negative (myostatin) regulators of fat browning [41,42]. In this regard, the crosstalk of leptin and irisin in the skeletal muscle and adipose tissue has provided further evidence to the exercise/leptin interaction. Leptin stimulates muscle accretion by increasing myocyte cell proliferation and myogenic factors (PGC-1α and myogenin) as well as by diminishing the expression of negative regulators of muscle growth including myostatin, dystrophin, or atrogenes MAFbx, or MuRF1 [36,43]. Moreover, leptin enhances the expression of the gene encoding irisin (*Fndc5*) through PGC-1α and iNOS-dependent mechanisms in the skeletal muscle and stimulates irisin-induced myogenesis, suggesting a synergic effect of both molecules on muscle growth [36]. In contrast, leptin downregulates FNDC5 expression in murine and human adipose tissue [36,44], and diminishes irisin-induced fat browning in murine adipocytes [43]. This inhibitory effect of leptin on irisin’s actions on adipocytes might constitute a compensatory mechanism to prevent energy depletion in the adipose tissue.

### 2.2. NO and Energy Balance

#### 2.2.1. Food Intake and Body Weight

Evidence suggests that NO may also be an intercellular modulator within the CNS, being involved in the control of energy homeostasis. In mice, the non-specific inhibition of NOS in the brain through the administration of Nω-Nitro-L-arginine methyl ester hydrochloride (L-NAME) decreases energy intake and body weight [45]. Moreover, diet-induced obese mice are more sensitive to the effects of non-specific NOS inhibition to reduce energy intake and body weight gain. Leptin deficient *ob*/*ob* mice exhibit elevated levels and activity of hypothalamic NOS, showing an increased sensitivity to the effects of NOS inhibition in food intake and body weight [46]. Furthermore, alterations in the fed state are associated with variations in the hypothalamic NOS levels [47], yielding further evidence of the role of NO in the regulation of energy balance. 

#### 2.2.2. Thermogenesis

BAT is found in direct contact with blood vessels in order to export heat to vital structures. The non-shivering thermogenic activity of BAT is evoked by norepinephrine (NE) secreted from the sympathetic nerves. The increased sympathetic activity in BAT in response to cold or high-fat diet induces vasodilation, which is mediated by the NO released from brown adipocytes [48]. Non-selective inhibition of NOS suppresses NE-induced increase in blood flow in a dose-dependent manner, confirming that NO controls BAT blood flow [49]. The sympathetic stimulation of rat brown adipocytes *in vivo* (i.e.; β_3_-adrenergic agonist or cold exposure) or *in vitro* (i.e.; noradrenaline treatment of cultured cells) confirm the presence of metabolically active eNOS and iNOS in the cytosol and nuclei. In genetically obese (*fa*/*fa*) rats, characterized by a marked reduction of BAT sympathetic activity, nuclear iNOS levels are lower than those of their lean counterparts [50]. Furthermore, the deletion of the iNOS gene reportedly decreases body weight as well as epididymal fat pads without changes in food intake, suggesting that *iNOS* knockout mice show an increased energy expenditure [51]. Moreover, an inhibitory effect of TNF-α, highly produced in obesity, on UCPs expression in a NO-dependent pathway involving iNOS expression has been described [52]. This mechanism could contribute to a decrease in energy expenditure. The absence of *iNOS* may reduce BAT NO production, thereby increasing the expression of UCPs and, hence stimulating the thermogenic activity. The increased expression of UCPs in BAT of *iNOS*-deficient mice has been described, confirming this mechanism [32].

### 2.3. Leptin, NO, and Energy Balance

#### 2.3.1. Food Intake and Body Weight

**Figure 1:**
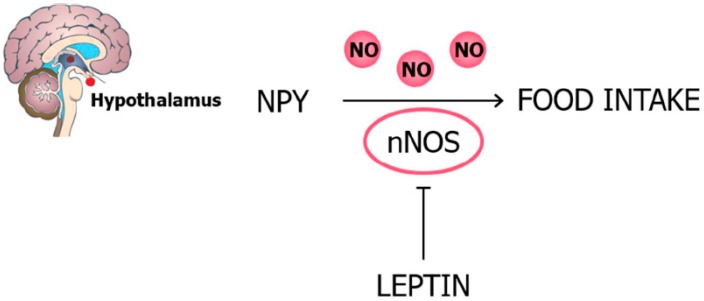
nNOS activity is required for the effects of leptin on food intake. Leptin alters the neuropeptide Y (NPY) effect decreasing nNOS activity by phosphorylation of the hypothalamic neurons of the ARC, DMH, and PMV. ARC, arcuate nucleus; DMH, dorsomedial nucleus of the hypothalamus; nNOS, neuronal nitric oxide synthase; PMV, ventral premammillary nucleus.

Leptin exerts its anorexigenic effects through the inhibition of the secretion of the orexigenic hypothalamic neuropeptides NPY and AgRP as well as the selective activation of POMC neurons. An induction of food intake by NPY mediated by NO has been shown. Moreover, leptin also alters NPY acting by decreasing nNOS activity through its phosphorylation in the hypothalamic neurons of the ARC, dorsomedial nucleus of the hypothalamus (DMH), and ventral premammillary nucleus (PMV) [53,54]. In this sense, *Nos1*-knockout mice exhibit a strong reduction in the appetite-suppressant activity of leptin [55]. Moreover, specific gene ablation of the *Lepr* in a subset of hypothalamic neurons expressing nNOS results in hyperphagia, obesity, hyperglycemia, and decreased energy expenditure, suggesting that the leptin-induced inhibition of the NO synthesis in the brain is involved in determining its effects on the central regulation of feeding behavior [56] (Figure 1).

#### 2.3.2. Thermogenesis

**Figure 2:**
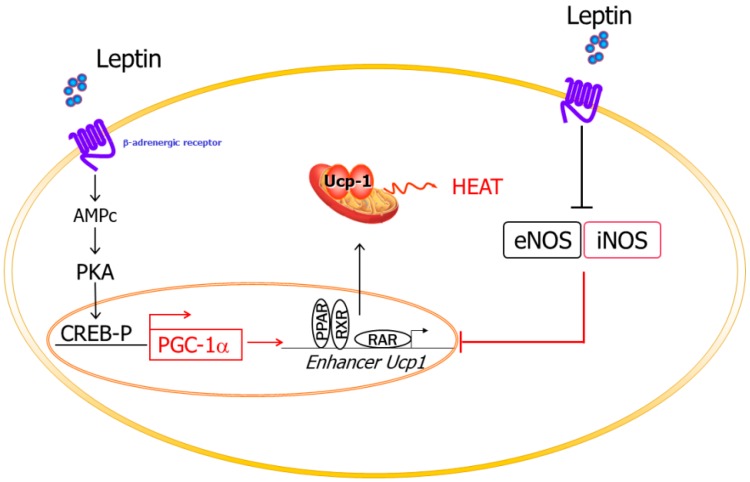
Leptin and NO participation in energy expenditure. Leptin increases energy expenditure by increasing UCP expression and through decreased NO production, which, in turn, activates the expression of UCP. NO, nitric oxide; UCP: uncoupling protein.

Leptin increases energy expenditure via the activation of the sympathetic activity, inducing the expression of UCPs, while NO downregulates the expression of UCPs in adipocytes, decreasing their thermogenic capacity. Moreover, leptin has been described to decrease the expression of different NOS isoforms in BAT [53]. Therefore, leptin increases energy expenditure by two mechanisms: (i) increasing the expression of the UCPs, and (ii) decreasing NO production, which, in turn, activates the expression of UCPs [52,53] (Figure 2). Furthermore, it has been described that the deletion of the *iNOS* gene decreases food efficiency through an increase in thermogenesis in the context of leptin-deficiency [32]. Nitric oxide is also implicated in brown-like transformation, participating in the recovery of the BAT phenotype and the improvement of brown fat cell function, likely involving the transcriptional coactivator mediator 1 (MED1) [57].

## 3. Glucose Metabolism

### 3.1. Leptin and Glucose Metabolism

Leptin plays a primary role in the regulation of glucose homeostasis through actions in the CNS and peripheral tissues beyond its effects on body weight and food intake [58].

At the CNS level, specialized subgroups of hypothalamic neurons exist that respond to changes in extracellular glucose concentrations. Leptin hyperpolarizes and inhibits their activity by the activation of ATP-sensitive K^+^ channels [59]. Leptin signaling, specifically in the hypothalamic ARC, is a major feeding-independent regulator of glucose homeostasis, improving hyperinsulinemia, and normalizing blood glucose levels. Therefore, the hypothalamus has been proposed as an important site for integration and regulation of energy homeostasis [60]. 

At the peripheral level, skeletal muscle is considered the major site of insulin- and exercise-stimulated glucose disposal. Leptin enhances the intracellular GLUT4 recruitment to the cell surface transport as well as glucose uptake in skeletal muscle [43]. Moreover, leptin also increases glucose uptake and enhances insulin signaling in skeletal muscle via the activation of sympathetic nerves and β_2_-AR [61]. Leptin also enhances the intracellular GLUT4 transport by reducing the expression and activity of the negative regulators of GLUT4 traffic TBC1D1 and TBC1D4 [62]. In the liver, leptin improves insulin sensitivity through complex effects on the hepatic gene expression of key metabolic enzymes and on intrahepatic partitioning of metabolic fluxes. Leptin administration inhibits the hepatic glucose production exerted by insulin [63]. At the same time, leptin increases gluconeogenesis and antagonizes some functions of insulin. However, the complex regulatory effect of leptin on the liver needs to be fully elucidated [64]. Compelling *in vitro* and *in vivo* evidence demonstrates that leptin inhibits both basal and glucose-stimulated insulin secretion in pancreatic β-cells. Moreover, leptin acutely downregulates preproinsulin mRNA expression in *ob*/*ob* mice and humans with obesity [65] as well as inhibiting pancreatic β-cell function [66]. In addition, leptin decreases insulin sensitivity and insulin-stimulated glucose uptake and incorporation into lipids in rodent adipocytes through different mechanisms [64]. 

A close relationship between leptin and other molecules in the glucose metabolism should not be discarded. Caveolin-1 (CAV-1), a 22 kDa integral membrane protein, is an essential protein constituent of membrane caveolae, particularly abundant in adipocytes and involved in insulin signaling [67]. An association between leptin and CAV-1 has been provided [68], suggesting an important role for CAV-1 in obesity and type 2 diabetes mellitus development.

### 3.2. NO and Glucose Metabolism

NO has emerged as a central regulator of energy metabolism mainly by modulating the oxidative capacity and insulin sensitivity of AT [37]. Genetically engineered mice lacking all three NOS isoforms developed nephrogenic diabetes insipidus, highlighting the relevant role of NO in maintaining glucose homeostasis [69].

iNOS is induced by inflammatory CK in skeletal muscle and fat, being involved in the pathogenesis of several metabolic disorders related with a low-grade chronic inflammatory state such as obesity-associated type 2 diabetes, characterized by insulin resistance [70,71]. The genetic disruption of iNOS expression protects against obesity-associated insulin resistance, suggesting that iNOS causes insulin resistance through NO-mediated tyrosine phosphorylation of key proteins involved in insulin signaling, promoting an impairment of glucose transport in liver and skeletal muscle [68,70]. Moreover, iNOS exhibits a pathogenic role in obesity-associated β-cell dysfunction and damage, inhibiting the glucose-stimulated secretion of insulin [72].

### 3.3. Leptin, NO, and Glucose Metabolism

Bradykinin, a NOS stimulator as well as NO, are specifically involved in the effect of leptin on glucose uptake in peripheral tissues. Leptin-induced muscle glucose uptake is blunted by NOS inhibitors, suggesting that NO production via bradykinin, activated by leptin, plays important roles in leptin-mediated glucose uptake in skeletal muscle, but not in AT [61]. In this sense, deletion of *iNOS* in leptin-deficient *ob*/*ob* mice improves hyperglycemia, hyperinsulinemia, and insulin resistance [32]. Interestingly, leptin action through neurons that express both OB-Rb and nNOS contributes slightly to the control of endocrine function, but is essential for the proper control of energy balance and glucose homeostasis, constituting the key site of action of the leptin-mediated control of systemic energy balance [56].

## 4. Lipid Metabolism

### 4.1. Leptin and Lipid Metabolism

Leptin directly participates in lipid metabolism control through the inhibition of lipogenesis and the stimulation of lipolysis, exerting an autocrine-paracrine lipolytic effect on isolated white adipocytes both *in vitro* and *ex vivo* [73,74,75]. This lipolytic effect is mediated by NO, without interfering with the catecholamine-mediated lipolysis [76]. Briefly, leptin leads to a suppression of lipogenesis and a regulation of fatty acid metabolism by reducing lipid synthesis as well as by partitioning fatty acids into oxidation rather than triacylglycerol storage [77]. In addition, leptin stimulates *Ucp2* mRNA expression and the Krebs cycle activity, contributing to fatty acid degradation as well as inhibiting fatty acid synthesis [78]. Different studies suggest that leptin prevents lipid storage and improves energy expenditure in WAT [77].

In liver, muscle, and pancreas, leptin also reduces lipid storage not only by promoting fatty acid oxidative pathways, but also by stimulating triacylglycerol hydrolysis. Chronic leptin administration also improves hepatic glycerol metabolism, an important metabolite for de novo TG synthesis in hepatocytes, by restoring the coordinated regulation of fat-specific aquaporin-7 and liver-specific aquaporin-9, a step that might prevent obesity-associated hepatosteatosis [79,80,81]. These actions avoid an excessive lipid accumulation and the development of different pathologies induced by lipotoxicity, since an excess of TG deposition in non-adipocytes cells leads to an impairment of their functions and an increased ceramide formation, triggering NO-mediated lipotoxicity and lipoapoptosis [82]. 

### 4.2. NO and Lipid Metabolism

The potential role of NO in the regulation of lipid metabolism has been demonstrated in several studies, confirming the expression and activity of eNOS and iNOS in AT [83]. Both *in vitro* and *in vivo* studies verify a NO-mediated effect on lipolysis regulation [84,85]. Lipolysis is under tight regulation by different hormones; being stimulated during fasting by increased levels of catecholamines and glucocorticoids, but being suppressed in the fed state by insulin action. In addition, there exists a huge number of potential modulators that also participate in its regulation. While chemical NO donors stimulate basal lipolysis *in vitro*, they mediate an inhibitory action on catecholamine-induced lipolysis via the inhibition of adenylyl cyclase (AC) and protein kinase A (PKA) [85,86]. These studies mostly involved the regulation of lipolysis by eNOS-release NO, with *eNOS*-knockout mice exhibiting increased abdominal fat mass, dyslipemia, and insulin resistance [87]. However, obesity is associated with increased iNOS expression in WAT, leading to an increased NO formation. NO produced by iNOS in obese individuals may impair insulin-stimulated glucose uptake or contribute to decreased lipolytic rates in subcutaneous AT, contributing to increased lipid storage and highlighting iNOS as a negative modulator of lipolysis via an oxidative signaling pathway upstream of cAMP production [88].

In liver, NO decreases hepatic lipogenesis through actions on coenzyme A and forming a metabolically inactive compound [89]. In skeletal muscle, NO also decreases lipogenesis via the activation of the AMP-activated protein kinase, associated with increased fatty acid oxidation during exercise [90]. Moreover, the treatment with L-NAME, a non-selective NOS inhibitor, upregulates the expression of PPAR-δ in skeletal muscle, a key transcription factor of fat oxidation in muscle cells. NO also affects the mitochondrial enzyme activity pattern, stimulating mitochondrial biogenesis and inhibiting fatty acid biosynthesis [91].

### 4.3. Leptin, NO, and Lipid Metabolism

The relationship between leptin action, NO, and lipid metabolism was provided for the first time by Frühbeck *et al*. [76], who observed a dose-dependent increase in both serum NO concentrations and basal adipose tissue lipolytic rate 1 h after exogenous leptin administration. Up to 27% of the variability taking place in lipolysis was caused by changes in NO concentrations. In fact, administration of the NOS inhibitor L-NAME prevented the leptin-induced release of glycerol, a marker of lipolysis, compared with leptin-treated control animals [76]. In contrast, the leptin-induced lipolytic effect was unaltered under pharmacologically acute ganglionic blockade. The NO donor S-nitroso-n-acetyl-penicillamine (SNAP) was able to exert a significant inhibitory effect on isoproterenol-stimulated lipolysis. These findings suggest that NO may function as an important autocrine-paracrine physiological regulator signal of leptin-induced lipolysis and, at the same time, is able to inhibit catecholamine-induced lipolysis. Leptin modulates NO production in WAT through protein kinase A (PKA) and mitogen-activated protein kinase (MAPK) activation. This mechanism may explain the positive correlation between plasma nitrite levels and fat mass [76].

**Figure 3:**
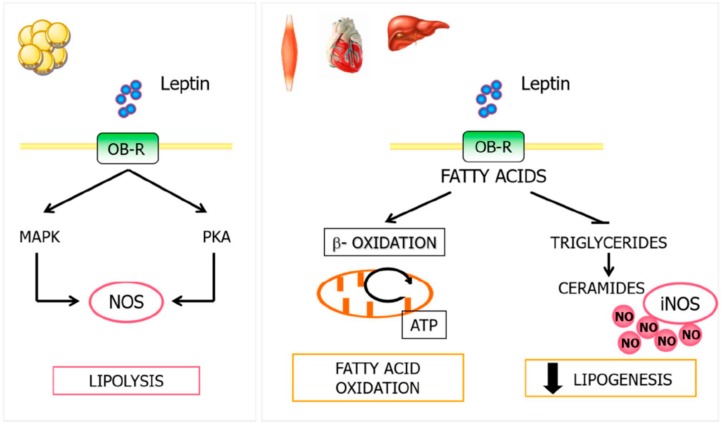
Relationship between leptin and NO in lipid metabolism. Leptin modulates NO production in WAT through PKA and MAP kinase activation, being an important regulator signal of leptin-induced lipolysis. In peripheral tissues, leptin induces fatty acid oxidation, inhibiting lipid accumulation, decreasing lipogenesis, and reducing lipotoxicity associated with non-oxidative fatty acid metabolism. MAPK, mitogen-activated protein kinase; NO, nitric oxide; PKA, protein kinase A; WAT, white adipose tissue.

Leptin also increases fatty acid oxidation in peripheral tissues including liver, heart, or skeletal muscle. The defective fatty acid oxidation related to obesity is associated with increased intracellular fatty acids, resulting in the activation of non-oxidative metabolic pathways and formation of ceramides. The excessive accumulation of ceramides increases the expression of iNOS and NO production, related to lipotoxicity, functional damage, and apoptosis [82]. Leptin favors fatty acid oxidation in peripheral tissues, inhibiting lipid accumulation, reducing lipogenesis, and decreasing lipotoxicity associated with non-oxidative fatty acid metabolism (Figure 3). Furthermore, leptin enhances NO production and lipid catabolism in human placenta [92].

## 5. Cardiovascular System

### 5.1. Leptin and the Cardiovascular System

#### 5.1.1. Angiogenesis and Wound Healing

Angiogenesis is defined as a biological mechanism of new blood vessel formation from pre-existing ones. This process plays important roles in many conditions including wound healing, menstrual cycles, or tumor development. Leptin is produced in tissues with high angiogenic activity including placenta, heart, and bone, defining leptin as a potent angiogenic factor involved in tumorigenesis, angiogenesis, and metastasis [67,93,94]. Leptin is co-expressed with the angiogenic mediator vascular endothelial growth factor (VEGF) and induces important angiogenic factors that can further increase VEGF expression, suggesting that leptin promotes neovascularization and modulates the angiogenic activity of VEGF in these tissues [95,96,97]. Consistent with this notion, leptin indirectly enhances the expression of the matrix metalloproteinase (MMP)-2 and MMP-9, contributing in the matrix remodeling of angiogenesis [98]. Furthermore, fibroblast growth factor (FGF)-2 is also essential for leptin-induced angiogenesis, producing synergistic effects with VEGF [97,99]. 

Leptin also participates in wound healing, in the neovascularization of damaged tissues as well as in the protection against acute gastric lesions [100,101]. Leptin-deficient mice exhibit impaired wound healing and leptin administration corrects this defect by accelerating proliferation, differentiation/function and migration of epidermal keratinocytes as well as enhancing angiogenesis around the wounded area [102,103].

#### 5.1.2. Vascular Health

High levels of leptin increase oxidative stress in endothelial cells, favor vascular smooth muscle cell migration and proliferation, reduce arterial distensibility, and contribute to obesity-associated hypertension [97,104,105]. All of these effects are inversely associated with vascular health and are deeply involved in the pathophysiology of atherosclerosis. However, its role in atherogenesis is not clear; leptin may exert pro- and anti-atherogenic effects depending on the dose of the adipokine used. Leptin administration within the subphysiological to physiological range dose-dependently reduces atherosclerotic disease indirectly by the attenuation of hypercholesterolemia and induction of adiponectin [106]. Moreover, increased plasma levels of leptin are independently associated with serum C-reactive protein concentrations, an acute marker of inflammation and a direct cause of cardiovascular diseases. These results strongly suggest that leptin may contribute to the pathophysiology of atherogenesis. Nevertheless, leptin is also suggested to be an important factor in the maintenance of vascular homeostasis and wall integrity [107]. Leptin-deficient mice exhibit atherosclerotic lesions and leptin administration reduces atherosclerosis through the reduction of hypercholesterolemia and liver steatosis as well as through the upregulation of the atheroprotective adiponectin [106,108]. The controversy about the involvement of leptin in cardiovascular diseases may be explained by the disparity between its beneficial and detrimental effects including the widespread cardiovascular and dose-dependent effects of leptin as well as the concept of selective leptin resistance.

#### 5.1.3. Blood Pressure

**Figure 4:**
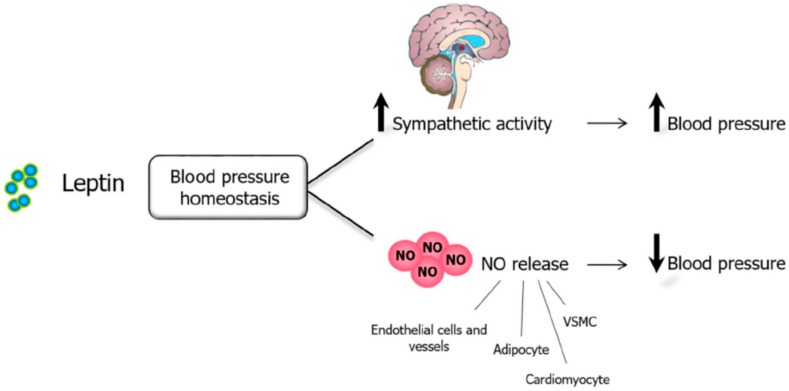
Leptin, NO, and blood pressure. Leptin exerts a dual influence on blood pressure control. Leptin activates sympathetic activity by centrally acting mechanisms, thus increasing blood pressure. However, direct leptin effects on peripheral tissues (endothelial cells, VSMC, adipocytes, cardiomyocytes, among others) lower blood pressure through increased NO release. NO, nitric oxide. VSCM, vascular smooth muscle cell.

Obesity was classified as a major risk factor for cardiovascular diseases by the American Heart Association in 1998 [109]. The actions of leptin in the cardiovascular system may help explain the link between excess body fat mass and cardiovascular diseases. Shortly after the discovery of leptin, investigators found a positive association between plasma leptin concentrations and sympathetic nerve activity. Short-term administration of leptin into the cerebral ventricles increases renal sympathetic activity, while long-term intravenous leptin infusions in nonobese rodents at rates similar to the levels found in severe obesity increase arterial pressure and heart rate through adrenergic activation [110]. Leptin exerts a biphasic effect on blood pressure regulation (Figure 4), a pressor response attributable to sympathoactivation [21,111] and, on the other hand, a depressor response attributable to the vasodilation of blood vessels, the increase of skeletal muscle insulin sensitivity, and the stimulation of natriuresis in kidney [21,112]. Our group was the first to identify NO in the leptin-induced effects on blood pressure [21]. Afterward, it was observed that leptin acts on the endothelium by stimulating the synthesis of NO via eNOS and nNOS activation through a PI3k independent activation of Akt and JAK2, respectively [113], or by increasing the NO production in vascular smooth muscle cells (VSMC) by activating iNOS [21,114]. Since leptin is able to stimulate NO release in both endothelial cells and vessels, the depressor response of this adipokine on blood pressure and vascular tone is attributable to a leptin-dependent NO release [21,114]. 

### 5.2. NO and the Cardiovascular System

#### 5.2.1. Angiogenesis and Wound Healing

NO plays a critical role in angiogenesis, mediating the proangiogenic response of significant factors including VEGF, angiopoetin-2, and estrogen [115]. These factors exert their effects by the activation of eNOS, the key isoform in neovascularization, providing a sustained flux of NO [116]. Furthermore, antiangiogenic agents such as endostatin and somatostatin mediate their effects through the activation of PP2A phosphatase, which is involved in the dephosphorylation of eNOS [89].

Furthermore, NO is critical in wound repair. Inflammatory cells present during the early phase of healing, especially macrophages and neutrophils, are responsible for most of the NO synthesis. The sustained NO synthesis is critical for wound collagen deposition and cell proliferation as well as for wound contraction [117,118]. Afterward, lower eNOS-derived NO levels become relevant during the proliferative and maturation phases [117]. In summary, NO plays a central role in the regulation of three major parts of the wound healing process: vascular homeostasis, inflammation, and antimicrobial action.

#### 5.2.2. Vascular Health

NO is a major regulator of vascular tone. Low levels of NO produced by eNOS in the endothelial cells maintain vasorelaxation by exerting their effects on VSMC. In addition to its vasorelaxing effects, NO inhibits the proliferation of VSMC, the aggregation of platelets, and the adherence and infiltration of inflammatory cells [119,120]. Moreover, arterial injury results in the upregulation of iNOS in VSMC in the arterial wall to prevent the adherence of platelets to the injured site and to preserve blood flow, playing a protective mechanism [120]. iNOS also exerts a protective role in the oxidative modification of LDL during the atherosclerosis process [121].

#### 5.2.3. Blood Pressure

NO plays a critical role in blood pressure regulation. The triple nNOS/eNOS/iNOS-null mice develop hypertension to a similar extent than eNOS-deficient mice, suggesting that eNOS constitutes the main form of NOS involved in the homeostasis of blood pressure [11,69]. NO produced in endothelial cells diffuses across VSMC membranes, promoting muscular relaxation and vasodilation. Furthermore, NO production also contributes to control blood pressure via the kidneys and the brain. Indeed, NO plays a key function in renal perfusion and glomerular filtration by affecting the afferent arteriolar tone [122]. In the brain, neuronal NO affects vasomotor tone and blood pressure by modulating the central sympathetic neural outflow [123]. In this regard, acute intravenous infusions of a nNOS-selective inhibitor, S-methyl-l-thiocitrulline, in healthy individuals increases systemic vascular resistance and blood pressure [124]. Impaired NO production or reduced NO bioavailability lead to vascular function alterations including hypertension [125].

### 5.3. Leptin, NO, and Cardiovascular System

#### Leptin, NO, and Blood Pressure

Leptin exerts a dual influence on blood pressure control, with its net effect depending on the balance between the pressor action due to the sympathoactivation and the vasorelaxant action due to the release of NO in endothelium, VSMC, and renal tubules. Leptin stimulates NO release from endothelial cells inducing vasodilation by directly activating a PI3K–independent Akt-eNOS phosphorylation pathway [113] as well as a JAK2-dependent nNOS induction [126]. Moreover, the endothelium-independent depressor action of leptin is mediated by the increased activation of iNOS in VSMCs via the activation of the JAK/STAT and PI3K/Akt pathway. The rise of NO is responsible for the inhibition of the leptin effect on the angiotensin II (Ang II)-induced vasoconstriction. The leptin-mediated NO increased in the vascular wall causes blood vessel vasodilation and attenuates the contractile response of VSMCs induced by Ang II through the attenuation of the Ang II-induced increase of cytosolic calcium [115] (Figure 4). Furthermore, the activation of JAK2/STAT3 and PI3K/Akt transduction signals in cardiomyocytes is related to the induction of iNOS by leptin, as NO is involved in the reduction of cardiac contraction [127]. Similar findings of the hypotensive effects of leptin via NO have been reported in rat myocardium [127], kidneys (through the activation of natriuresis and diuresis [128]), endothelium of conduit vessels [119], mesenteric and coronary arteries [129] as well as perivascular AT, supporting the idea that NO represents a key mediator of the cardiovascular effects of leptin. 

### 5.4. Leptin, NO, and Angiogenesis

Leptin promotes angiogenesis, which is regulated by different factors promoting neovascularization including VEGF, platelet derived growth factor (PDGF), or NO [130]. Furthermore, NO mediates the proangiogenic response of different key factors including VEGF, estrogens, or angiopoetin-2 [131]. Since leptin induces eNOS activity, which increases vasodilation and blood perfusion necessary in angiogenesis, an angiogenic effect of leptin through NO could be inferred.

A close relationship between angiogenesis and adipogenesis has been identified. During obesity, the expansion of fat mass is associated with hypoxia, an important factor for vascular growth and extracellular matrix remodeling. In response to hypoxia, AT releases hypoxia inducible factor 1 (HIF-1), induces angiogenic factors including VEGF, leptin, or TNF-α, which are involved in the regulation of angiogenesis and vasculogenesis [95,132]. Moreover, the inflammatory response that emerges in the presence of obesity is associated with the recruitment of inflammatory cells. These activated macrophages induce the synthesis of angiogenic factors such as TNF-α, IL-6, IL-8, or NO, contributing to adipose neovascularization. The activation of iNOS by leptin is reportedly necessary for the synthesis and secretion of tenascin C in both adipocytes and hepatocytes, suggesting an important role of this alarmin in the development of AT as well as hepatic inflammation and fibrosis, processes closely linked to angiogenesis [133].

Abnormalities or defects in the physical properties of the cell membranes may be linked to hypertension, stroke, and other cardiovascular diseases. Leptin actively participates in the regulation of membrane properties, increasing the membrane fluidity, and improving the microviscosity of erythrocytes [119]. The effect of leptin on the membrane fluidity is significantly potentiated by NO donors and attenuated in the presence of NOS inhibitors, suggesting that leptin increases the membrane fluidity and improves the rigidity of the cell membranes through NO-dependent pathways [119]. Since NO is involved in the regulation of subcutaneous AT blood flow in humans, it is tempting to speculate that an improvement in erythrocyte membrane microviscosity could be modulated via leptin-induced NO production.

## 6. Bone Metabolism

### 6.1. Leptin and Bone Metabolism

Leptin exerts dual effects depending on bone tissue, skeletal maturity, and/or the signaling pathway. Early in life, leptin stimulates bone growth through direct angiogenic and chondro-osteogenic effects, enhancing osteoblastic proliferation, de novo collagen synthesis, and mineralization [134] as well as inhibiting osteoclastogenesis [135]. The leptin receptor is expressed in adult primary osteoblasts and chondrocytes, suggesting that these effects of leptin on bone metabolism may be direct [7]. Later, in the mature skeleton, leptin acts through indirect mechanisms, by central (hypothalamic) pathways, decreasing bone remodeling and formation [136]. The hypothalamic negative effects could compensate the peripheral and positive ones that predominate only when central leptin resistance occurs with obesity onset or else when the serum leptin level rises above a certain threshold [137,138]. In this sense, leptin acts as a direct skeletal growth factor in obese children, characterized by differential sensitivity to circulating leptin and central leptin resistance [139]. Moreover, the increase in bone mineral density observed in obese humans might be secondary to leptin resistance, critical in human obesity development. However, although leptin alters bone metabolism, conclusions on the nature of these effects remain highly controversial so far.

### 6.2. NO and Bone Metabolism

NO is an important signaling molecule in bone, and mediates the function of bone cells as well as the process of bone remodeling [140]. Low concentrations of NO increase bone loss with CK being necessary for normal osteoclast function and stimulating bone resorption [141]. Nevertheless, high concentrations of NO inhibit bone resorption by suppressing the production of osteoclasts and their activity. Furthermore, the growth and differentiation of osteoblasts are inhibited by higher concentrations of NO [142,143]. *iNOS*-knockout mice exhibit profound defects in osteoclastic bone resorption and reduced inflammation-induced osteoporosis, suggesting that the iNOS isoform is responsible for the bone loss associated with inflammatory processes [142,144].

### 6.3. Leptin, NO, and Bone Metabolism

**Figure 5:**
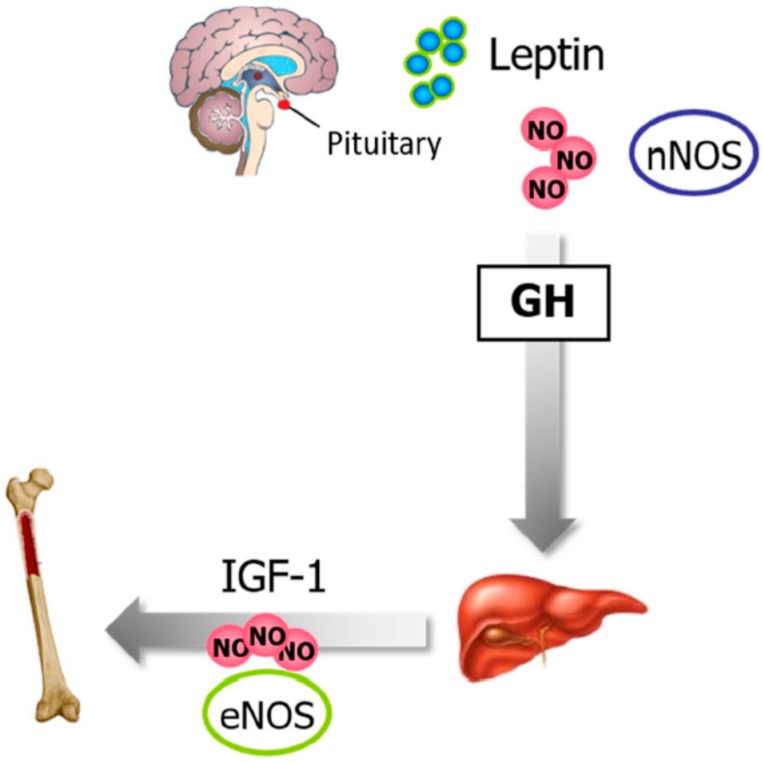
Leptin, NO, and bone metabolism. Leptin directly influences GH regulation and secretion via the activation of pituitary nNOS. GH stimulates skeletal growth by inducing liver production of IGF-1, which in turn, via NO, stimulates osteoblastic activity. GH, growth hormone; IGF-1, insulin-like growth factor 1; NO, nitric oxide.

The growth hormone (GH) is secreted by the pituitary and acts on peripheral tissues through the stimulation of insulin-like growth factor 1 (IGF-1) synthesis and secretion. Indeed, GH and IGF-1 axis have pleiotropic effects on the skeleton by influencing bone formation and resorption, with GH stimulating osteoblastogenesis and bone formation. Leptin directly influences GH regulation and secretion via the activation of nNOS, with NO being necessary to induce GH secretion [145,146]. GH stimulates skeletal growth by inducing liver production of IGF-1, which in turn stimulates osteoblastic activity in an endocrine manner in order to promote skeletal growth. The IGF-1 effects may be partly mediated by increased NO release from the endothelium (Figure 5). *eNOS*-knockout mice exhibit bone defect formation due to the impaired IGF-I response [147].

## 7. Reproduction

### 7.1. Leptin and Reproduction

Leptin-deficient female mice are infertile with leptin treatment restoring their fertility, thereby demonstrating its significance in reproduction. Leptin plays an integral role in the physiology of the reproductive system with complex interactions at all levels of the hypothalamic-pituitary gonadal axis (HPG). Thus, leptin serves as a putative signal that links metabolic status with the reproductive axis [148]. At the hypothalamic level, leptin mediates the pulsatile release of gonadotropin-releasing hormone (GnRH), thus controlling the secretion of the pituitary gonadotropins luteinizing hormone (LH) and follicle-stimulating hormone (FSH). Moreover, leptin induces a dose-related increase pituitary prolactin release [19]. As leptin is influenced by steroid hormones and can stimulate LH release, it has been hypothesized that leptin acts as a permissive factor in the development of puberty [149]. In ovaries, leptin acts directly on granulosa cell steroidogenesis, promoting follicular development and oocyte maturation, with important implications for ovulation induction [150]. In the endometrium, leptin plays an important role in embryonic development and implantation, and acts as a homeostatic regulator in the placenta [151].

In type A spermatogonial stem cells, leptin may act to prevent differentiation, allowing the cells to undergo stem cell renewal, whereas in spermatocytes, leptin may assist the cells through full differentiation and maturation to spermatids. 

### 7.2. NO and Reproduction

NO is involved in sexual and reproductive function at every level in the organism. In this regard, the triple nNOS/eNOS/iNOS-null mice exhibit a marked reduction in fertility rates [69]. In the brain, NO induces the release of LH, which reaches the pituitary gland, activating the release of gonadotropins through nNOS activation [152]. NO is synthesized in ovules, being involved in ovulation, luteolysis, steroidogenesis, apoptosis of follicular cells, the oocyte meiosis, and in the regulation of the oocyte transfer from the ovaries to the oviducts, among others [153,154,155]. Furthermore, NO may play an important role in the endometrial receptivity, implantation, and menstruation [156,157]. In the testis, NO also regulates spermatogenesis, sperm maturation, and mobility as well as the apoptosis process in the abnormal germ cells [158]. Moreover, NO decreases sperm motility, inducing sperm toxicity and infertility, constituting a physiologic signal essential to penile erection.

### 7.3. Leptin, NO, and Reproduction 

Leptin regulates the delivery of GnRH at the hypothalamic level, promoting the secretion of LH and FSH in a pulsatile fashion together with prolactin in the pituitary. This adipokine coordinates the reproductive function by acting on neurons in the preoptic region of the hypothalamus and inducing the synthesis of NO through the activation of nNOS in these neurons. NO acts as a mediator of leptin signaling to the central reproductive axis [159]. Targeted deletion of the leptin receptor in nNOS neurons has a profound effect on energy balance, providing evidence that nNOS neurons are crucial in the integration of metabolic signals. Furthermore, genetic deletion as well as pharmacological inhibition of nNOS prevented the stimulatory effects of leptin on GnRH/LH secretion [160].

Leptin also plays a role in enhancing the fertilization capacity of human spermatozoa by increasing motility and acrosome reaction through the increase of NO production via PI3K [161]. Moreover, leptin regulates NO concentrations during the formation of the placenta and the embrionary organogenesis [92].

## 8. Immune Response

### 8.1. Leptin and the Immune Response

Leptin regulates both innate and adaptive responses through the modulation of immune cell survival and proliferation as well as its activity. Accordingly, OB-Rb is expressed in the cells of both the innate and adaptive immune system, linking nutritional status and immune function. Administration of leptin reverses the immunosuppressive effects of acute starvation in mice [162], and obese leptin-deficient *ob*/*ob* mice exhibit defective immune responses, partly reversed after leptin administration [163]. 

#### 8.1.1. Leptin and Hematopoiesis

The leptin receptor is identified in yolk sac, fetal liver, bone marrow as well as in lympho-hematopoietic, fetal stromal, and megakaryocytic cell lines, suggesting a role for leptin in hematopoiesis [164,165,166]. Colony-forming assays demonstrate that leptin treatment increases hematopoietic stem cell proliferation, directly stimulating both the myeloid and lymphoid lineages. However, although data about the role of leptin in the direct regulation of hematopoietic cell proliferation might be contradictory, *in vivo* results strongly suggest a plausible indirect regulatory role for leptin in hematopoiesis, particularly on the lymphocytic lineage. Leptin receptor-deficient mice exhibit a defective erythrocyte production in the spleen [166], whereas a decrease in the number of circulating lymphocytes and an increase in monocytes is observed in *ob*/*ob* mice [150].

#### 8.1.2. Leptin and the Innate Immune Response

**Figure 6:**
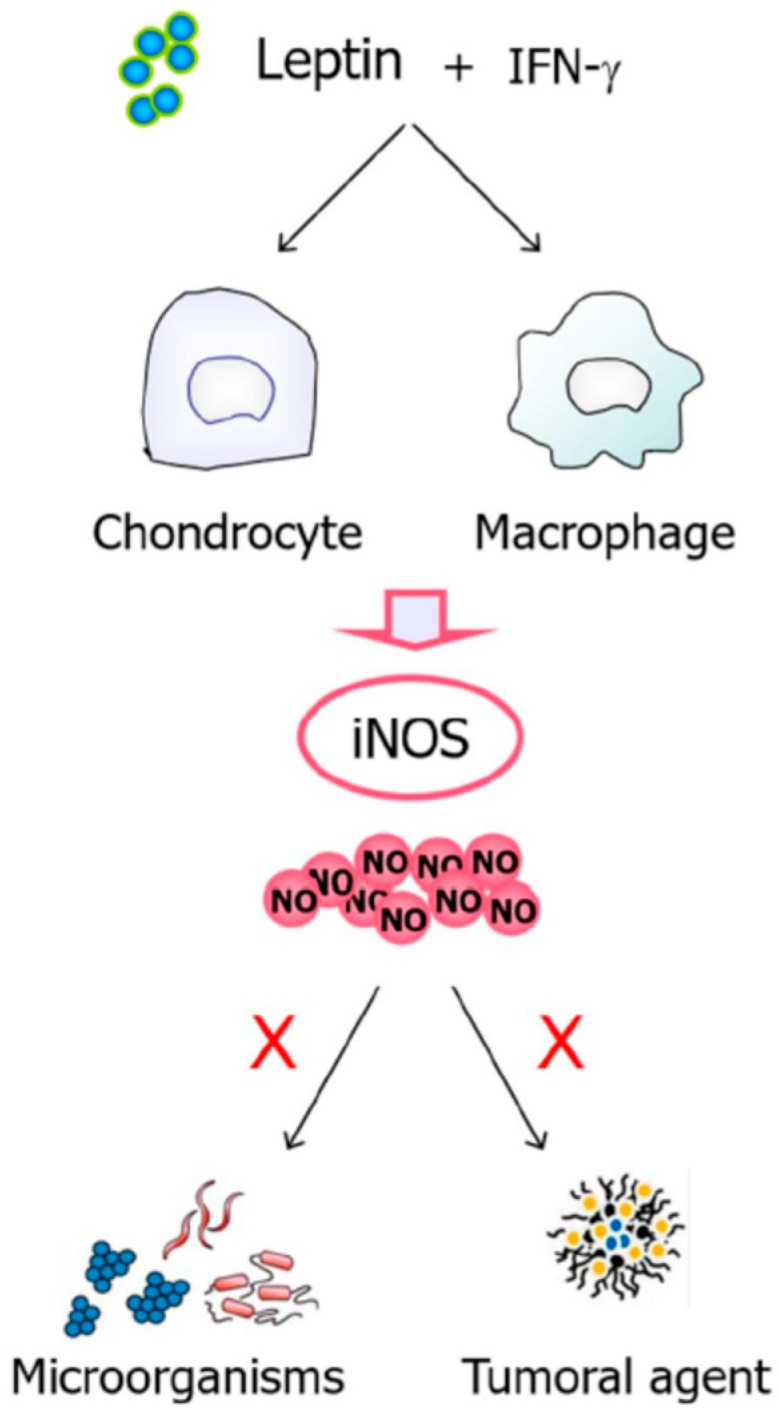
Leptin acts through NO in the immune response. Leptin markedly increases interferon-γ (IFN-γ)-induced NO production by increasing iNOS and COX-2 expression in macrophages and chondrocytes, playing a proinflammatory role in synergy with interleukin (IL)-1 and maintaining an inflammatory response. NO is responsible for the destruction of microorganisms and tumoral agents. Moreover, leptin stimulates GH production by peripheral blood mononuclear cells via NO-dependent pathways. COX-2, cyclooxygenase 2; GH, growth hormone, iNOS, inducible nitric oxide synthase; NO, nitric oxide.

The innate response is considered the first line of the immune defense, being activated by the presence of antigens and their chemical properties. It is comprised of physical, chemical, and biological barriers as well as specialized cells and soluble molecules. The increased sensitivity of *ob*/*ob* mice to proinflammatory and monocyte/macrophage-activating stimuli suggests a role for leptin in the regulation of inflammatory responses. Leptin is involved in the proinflammatory response through the activation of macrophages/monocytes that in turn upregulates the phagocytic function via phospholipase activation as well as the secretion of proinflammatory CK including TNF-α (early), IL-6 (late), and IL-12 [167,168]. Furthermore, leptin induces the production of factors involved in the regulation of the immune response including leukotriene B4, eicosanoids, cyclooxygenase 2 (COX-2), or NO (Figure 6). In neutrophils, leptin increases chemotaxis and the release of oxygen radicals [169]. Moreover, the adipokine promotes proliferation and activation of natural killer cells, increasing their cytotoxicity, and also regulates the M1- to M2-phenotype polarization [170]. Finally, leptin is involved in dendritic cell maturation and survival [171].

#### 8.1.3. Leptin and the Adaptive Immune Response

In adaptive immunity, leptin increases the proliferation of naïve T and B cells while it reduces that of regulatory T cells (Treg), stimulating the IL-2 expression. Furthermore, leptin promotes the switch toward pro-inflammatory Th1 cells (which secrete the proinflammatory CK interferon γ) rather than the anti-inflammatory Th2 phenotype (characterized by the secretion of IL-4 and IL-10), and facilitates Th17 responses. Finally, leptin activates B cells to secrete proinflammatory CK and modulates B cell development. Leptin also enhances the expression of adhesion molecules including ICAM-1, involving lymphocyte migration toward inflammatory sites [162]. Leptin has been shown to affect thymic homeostasis, protecting thymocytes from apoptosis as well as accelerating their maturation. In addition, the product of the *ob* gene has anti-apoptotic effects on mature T cells and hematopoietic precursors [172].

#### 8.1.4. Leptin and Autoimmunity

Elevated levels of circulating leptin have been associated with multiple autoimmune diseases including type 1 diabetes, multiple sclerosis, or rheumatoid arthritis [173].

### 8.2. NO and the Immune Response 

NO is important as a toxic defense molecule against infectious organisms, as an apoptotic inducer, and as a critical immunoregulatory player by upregulating macrophage-derived proinflammatory CK. The generation of NO is a feature of genuine immune-system cells (dendritic cells, natural killer cells, mast cells, and phagocytic cells including monocytes, macrophages, microglia, Kupffer cells, eosinophils, and neutrophils) as well as of cells involved in immune reactions [174]. 

#### 8.2.1. NO and Hematopoiesis

NO may act as a potent antiproliferative factor in a variety of cells, contributing to the homeostasis of the stem cell pool. Expression of various NOS isoforms has been described in human and rodent bone marrow (BM) and blood. Inhibition of NOS activity *in vivo* is associated with an increase in the number of stem and early progenitor cells in the BM, together with an increase in the neutrophil content in the blood [175]. Specifically, the nNOS isoform expressed in stromal cells acts as a paracrine regulator of hematopoietic stem cells. The inactivation of the *NOS1* gene increases the number of cell colonies that can be generated from the bone marrow and the spleen [176]. In addition, the eNOS expressed by BM stromal cells influences the recruitment of stem and progenitor cells [177].

#### 8.2.2. NO and Innate Immune Response

The NO produced by iNOS in mammalian macrophages in response to inflammatory stimuli such as lipopolysaccharide or CK (IL-1β, TNF-α, IFN-γ) constitutes an essential component of the host immune response against foreign organisms including bacteria, parasites, and viruses [178]. On the one hand, NO exerts a direct microbicidal effect via the reaction of NO with iron or thiol groups on proteins forming iron–nitrosyl complexes that inactivate crucial enzymes in mitochondrial respiration or DNA replication of pathogens. On the other hand, NO may react with superoxides to form reactive oxidants capable of damaging target cells by lipidic peroxidation in cellular membranes [179]. Furthermore, NO can also induce macrophage apoptosis or stimulate their cytoplasmic motility as well as modulate neutrophil adhesion and the differential regulation of cytokine synthesis by leukocytes [180]. NO may efficiently combat infections, but alterations in pro-and anti-inflammatory CK may be responsible for immune-mediated pathologies or affect normal physiological functions of NO [181].

#### 8.2.3. NO and the Adaptive Immune Response

NO is also involved in the control of the adaptive immune response through the regulation of adaptive immune cell induction and function. Low concentrations of NO have a selective enhancing effect on the induction and differentiation of Th1, but not Th2 cells, via the induction of the expression of IL-12 receptor β2 [182]. Th1 cells characteristically produce IFN-γ, which can strongly activate macrophages to produce high concentrations of NO via iNOS, whereas Th2 cells produce IL-4 and IL-5, which can inhibit iNOS induction. High NO concentrations inhibit IL-12 production, limiting the differentiation of Th1 cells in order to avoid an excess of this type of cells and contributing importantly to immune homeostasis [182]. The balance between Th1 and Th2 cells may determine the progression of many infections and autoimmune diseases. 

#### 8.2.4. NO and Autoimmunity

Protein expression levels of iNOS have been found in the majority of autoimmune diseases, mostly in inflammatory cell infiltrates such as activated macrophages, but also in organ-specific epithelial cells or in parenchymal cells, with NO playing an important role in the pathogenesis of autoimmune disorders [183,184].

### 8.3. Leptin, NO, and Immune Response

NO mediates many effects of leptin in the proinflammatory response. Leptin markedly increases IFN-γ-induced NO production in a concentration-dependent manner by increasing iNOS and COX-2 expression in macrophages [185] and chondrocytes [186], contributing to sustain the ongoing inflammatory response. The NO released is responsible for the destruction of microorganisms and tumoral agents. Furthermore, leptin stimulates the production of the GH by peripheral blood mononuclear cells (PBMC) via a NO-dependent pathway, which is responsible for the migration of fresh and activated lymphocytes and the increase of T cell adhesion [168]. 

## 9. Obesity

### Leptin, NO, and Obesity

Obesity constitutes a chronic low-grade inflammatory condition, with the WAT being the major site for the initiation and exacerbation of this condition, undergoing a continuous process of remodeling [187]. Adipocyte hypertrophy and hypoxia due to adipocyte expansion are the two main contributing factors to the increased accumulation of macrophages in WAT in the obese state. Macrophages are phenotypically modified toward a proinflammatory M1 phenotype, promoting the expression of inflammatory mediators including iNOS [188]. Moreover, leptin is an important factor of the innate and adaptive immune system that mediates an inflammatory response by regulating the production of proinflammatory cytokines, favoring the chronic proinflammatory state [172]. Our group has shown that the activation of iNOS by leptin in AT is necessary for the synthesis and secretion of the profibrogenic and proinflammatory tenascin C, an important glycoprotein involved in the etiopathology of obesity via AT inflammation and fibrosis [133,189].

Leptin is also a key regulator for inflammation and progression of fibrosis in various chronic liver diseases including non-alcoholic steatohepatitis. Leptin action in macrophages of the steatotic liver, through induction of iNOS and NADPH oxidase, causes peroxynitrite-mediated oxidative stress thus activating Kupffer cells, promoting inflammation in the fatty liver [190]. Furthermore, iNOS activation induced by leptin is needed and crucial for the synthesis and release of tenascin C, which is also involved in the development of hepatic inflammation and fibrosis [191].

Furthermore, it has been described that leptin enhances the inductive action of IL-1 and IL-8 on iNOS, and thus on NO production, contributing to an increased risk of osteoarthritis in obesity [192]. 

## 10. Conclusions

NO is a fundamental signal for the action of leptin in all types of tissues including adipocytes, neurons, immune cells, muscle, or β-cells. Since the product of the *ob* gene exerts its effects on the CNS and peripheral tissues, the NO mediated role of leptin in cell physiology is complex, and depends on the cell type, the NOS isoform, or the NO concentration, with leptin also playing a crucial role in the metabolism of NO. Although further studies should be conducted to more thoroughly explore the relationship between NO and leptin, the leptin–NO axis is reportedly involved in the pathophysiology of several diseases.

## Abbreviations

AC, Adenylyl cyclase; AgRP, agouti gene-related protein; Ang, angiotensin; AR, adrenergic receptor; ARC, arcuate nucleus; AT, adipose tissue; BAT, brown adipose tissue; CART, cocaine-amphetamine-regulated-transcript; CK, cytokine; CNS, central nervous system; CRH, corticotropin-releasing hormone; DMH, dorsomedial nucleus of the hypothalamus; EDRF, endothelial-derived relaxing factor; eNOS, endothelial nitric oxide synthase; FSH, follicle-stimulating hormone; GH, GnRH, gonadotropin-releasing hormone; growth hormone; HPG, hypothalamus-pituitary-gonads; ICV, intracerebroventricular; IFN-γ, interferon-γ; IL, interleukin; iNOS: Inducible nitric oxide synthase; LH, luteinizing hormone; L-NAME, Nω-Nitro-l-arginine methyl ester hydrochloride; MCH, melanin-concentrating hormone; MMP, metalloproteinase; NE, norepinephrine; NO, nitric oxide; NPY, neuropeptide Y; nNOS, neuronal nitric oxide synthase; Pgc-1α, peroxisome proliferator-activated receptor γ coactivator-1 α; PKA, protein kinase A; POMC, proopiomelanocortin; Ppar-δ, peroxisome proliferator-activated receptor-δ; PVN: paraventricular nucleus; SNAP, S-nitroso-n-acetyl-penicillamine; Tnf-α, tumor necrosis factor-α; UCP-1, uncoupling protein 1; VEGF, vascular endothelial growth factor; VSMC, vascular smooth muscle cells; VTA, ventral tegmental area; WAT, white adipose tissue.
